# Supplementary material for: Catalytic activity imperative for nanoparticle dose enhancement in photon and proton therapy
Source: Nat Commun. 2022 Jun 6;13:3248. doi: 10.1038/s41467-022-30982-5 (PMC9170699; doi:10.1038/s41467-022-30982-5)
Supplement: Supplementary file 1 — Supplementary Information [file 41467_2022_30982_MOESM1_ESM.pdf]

Supplementary Information

Catalytic activity imperative for nanoparticle dose enhancement in photon and proton therapy

Lukas R.H. Gerken,[1,2] Alexander Gogos,[1,2] Fabian H.L. Starsich,[1,2] Helena David,[1] Maren E. Gerdes,[1] Hans Schiefer,[3] Serena Psoroulas,[4] David Meer,[4] Ludwig Plasswilm,[3,5] Damien C. Weber[4,5,6] and Inge K. Herrmann[1,2,]*

[1] Nanoparticle Systems Engineering Laboratory, Institute of Energy and Process Engineering (IEPE), Department of Mechanical and Process Engineering (D-MAVT), ETH Zurich, Sonneggstrasse 3, 8092 Zurich, Switzerland.

[2] Particles-Biology Interactions Laboratory, Department of Materials Meet Life, Swiss Federal Laboratories for Materials Science and Technology (Empa), Lerchenfeldstrasse 5, 9014 St. Gallen, Switzerland.

[3] Department of Radiation Oncology, Cantonal Hospital St. Gallen (KSSG), Rorschacherstrasse 95, CH-9007 St. Gallen, Switzerland.

[4] Center for Proton Therapy, Paul Scherrer Institute, Forschungsstrasse 111, 5232 Villigen PSI, Switzerland.

[5] Department of Radiation Oncology, Inselspital University Hospital, 3010 Bern, Switzerland.

[6] Department of Radiation Oncology, University Hospital Zürich, 8091 Zürich, Switzerland.

*ingeh@ethz.ch

Supplementary Table 1: Atom composition and density of selected nanoparticles.

Nanoparticle	Theoretical Atom Composition			Atomic number	Bulk Density
	X	(mol)	(wt%)	Z	(g/cm^3)
SiO_2	Si<br>O	1/3<br>2/3	46.7<br>53.3	14<br>16	2.7
TiO_2	Ti<br>O	1/3<br>2/3	49.9<br>50.1	22<br>16	4.3
TiN	Ti<br>N	1/2<br>1/2	77.4<br>22.6	22<br>7	5.4
WO_3	W<br>O	1/4<br>3/4	79.3<br>20.7	74<br>16	7.2
HfO_2	Hf<br>O	1/3<br>2/3	84.8<br>15.2	72<br>16	9.7
Au	Au	1	1	79	19.2

X: Chemical symbol.

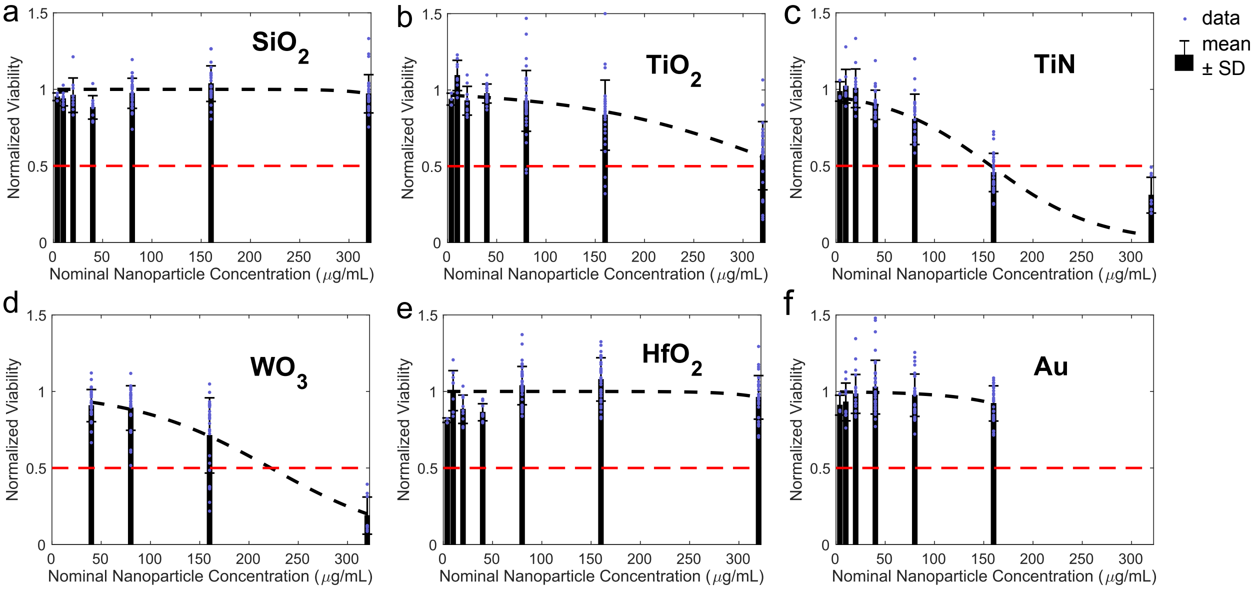

Supplementary Figure 1: Effect of 24-hour nanoparticle treatment on long term cell viability. Sham-irradiation (0 Gy) viability of HT1080 cells incubated with SiO_2 (a), TiO_2 (b), TiN (c) WO_3 (d), HfO_2 (e) and Au (f) nanoparticles. Red dashed line indicates 50% viability. Normalized viability is the surviving fraction of cells compared to the control cells (no nanoparticles) 5 days after sham irradiation with X-rays or protons. Bars expressed as mean ± SD from individual data points (grey dots) collected over 6 independent experiments.

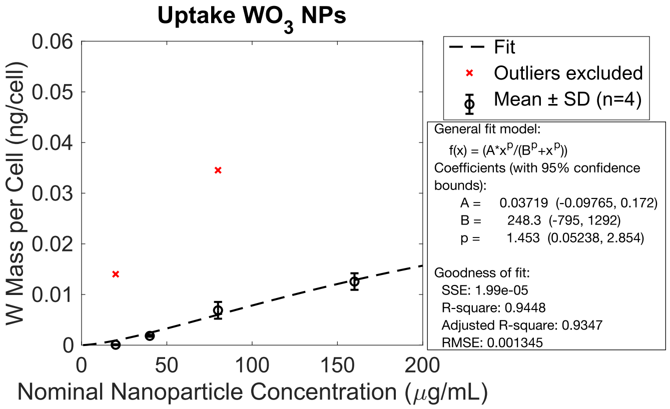

Supplementary Figure 2: Cellular uptake quantification of WO_3 nanoparticles. Metal mass of Tungsten per cell as quantified using ICP-MS. Data given as mean ± SD from n = 4 biological replicates examined over 2 independent experiments.

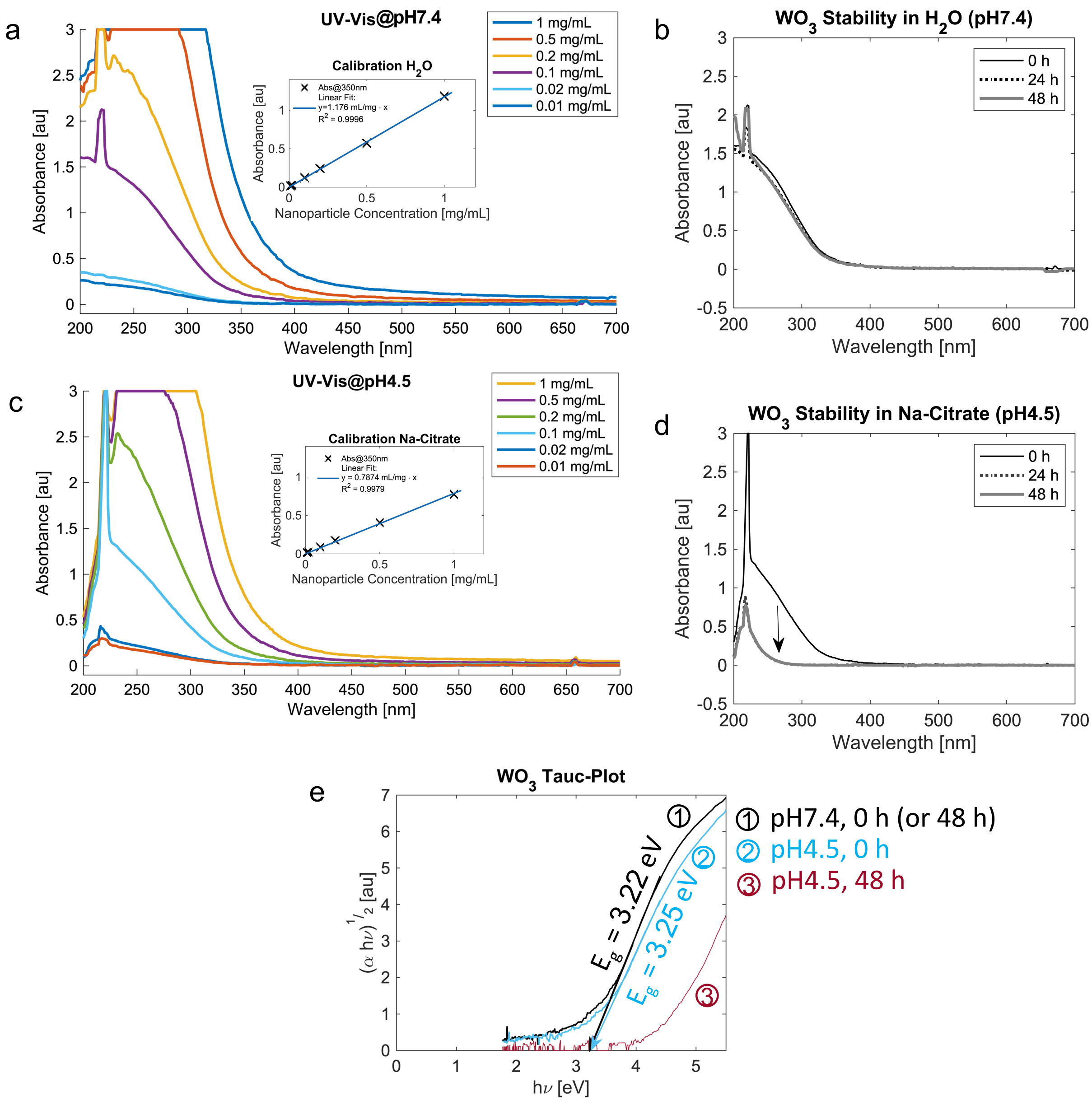

Supplementary Figure 3: Dissolution of FSP synthesized WO_3 nanoparticles in lysosome-mimicking, acidic Na-citrate buffer (pH 4.5), but not in Milli-Q water (pH 7.4), analyzed using UV–Vis (Jenway 6705, Cole-Parmer, Staffordshire, UK) absorbance measurements. Absorbance spectrum in water (a) and Na-citrate buffer (b); inset showing the linear calibration curve for the absorbance at 350 nm and at different nanoparticle concentrations. Absorbance spectrum for 0.1 mg/mL nanoparticle concentration in water (c) and Na-citrate buffer (d) after 0, 24 and 48 hours. Tauc-plot (e) showing the characteristic band gap of the WO_3 nanoparticles at 0 hours (3.22-3.25 eV), which is non-existent after several hours in Na-citrate buffer (exemplarily shown for the 48h interval).

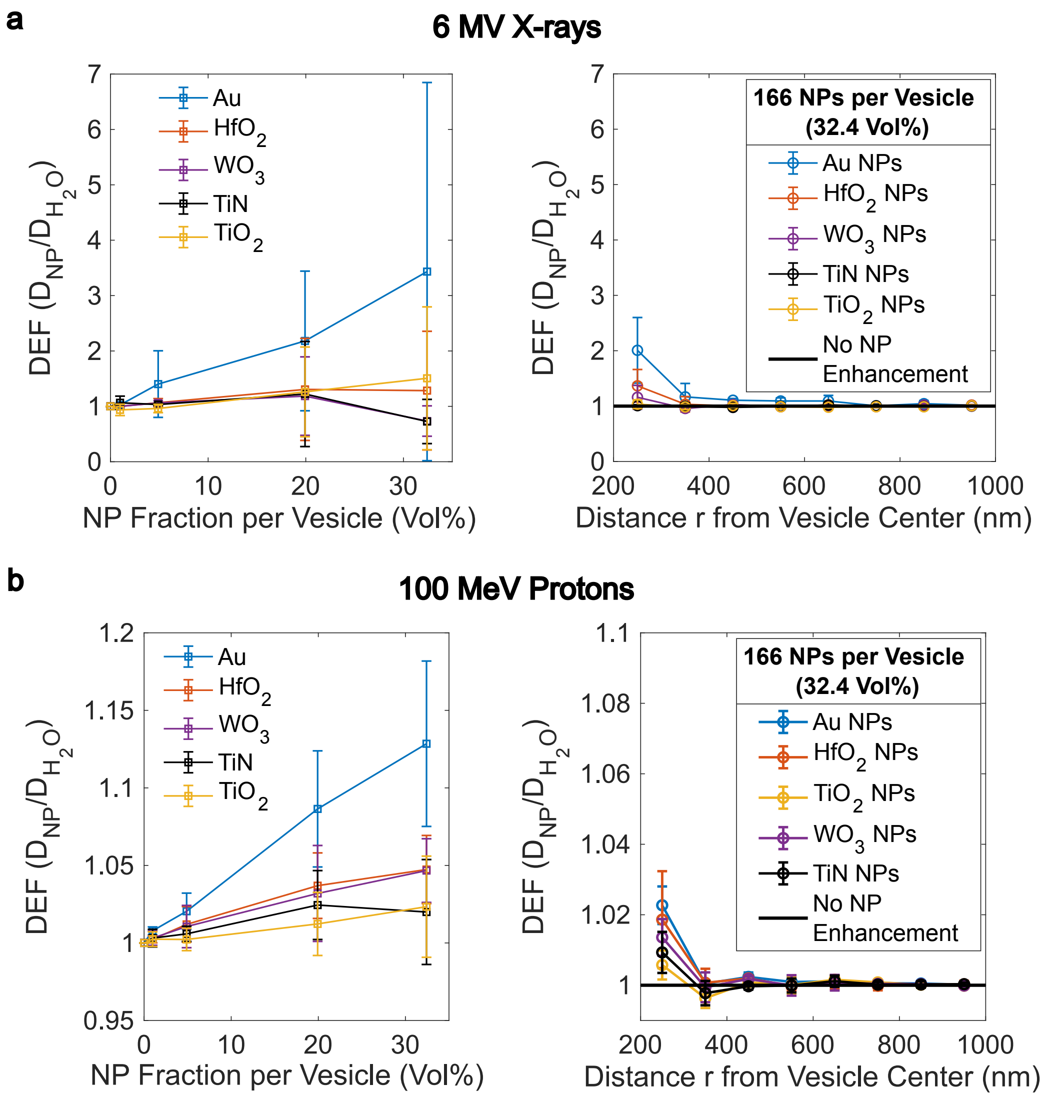

Supplementary Figure 4: Nanoscale dose enhancement of nanoparticles for high energy photons or protons. Physical Dose Enhancement Factors (DEF) inside (left) and around (right) 400-nm sized, nanoparticle-filled vesicles for 50 nm sized Au, HfO_2, WO_3, TiN and TiO_2 nanoparticles and 6 MV X-ray (a) or 100 MeV proton irradiation (b). Data given as mean ± SD from N = 3 simulation experiments. Source data are provided as a Source Data file.

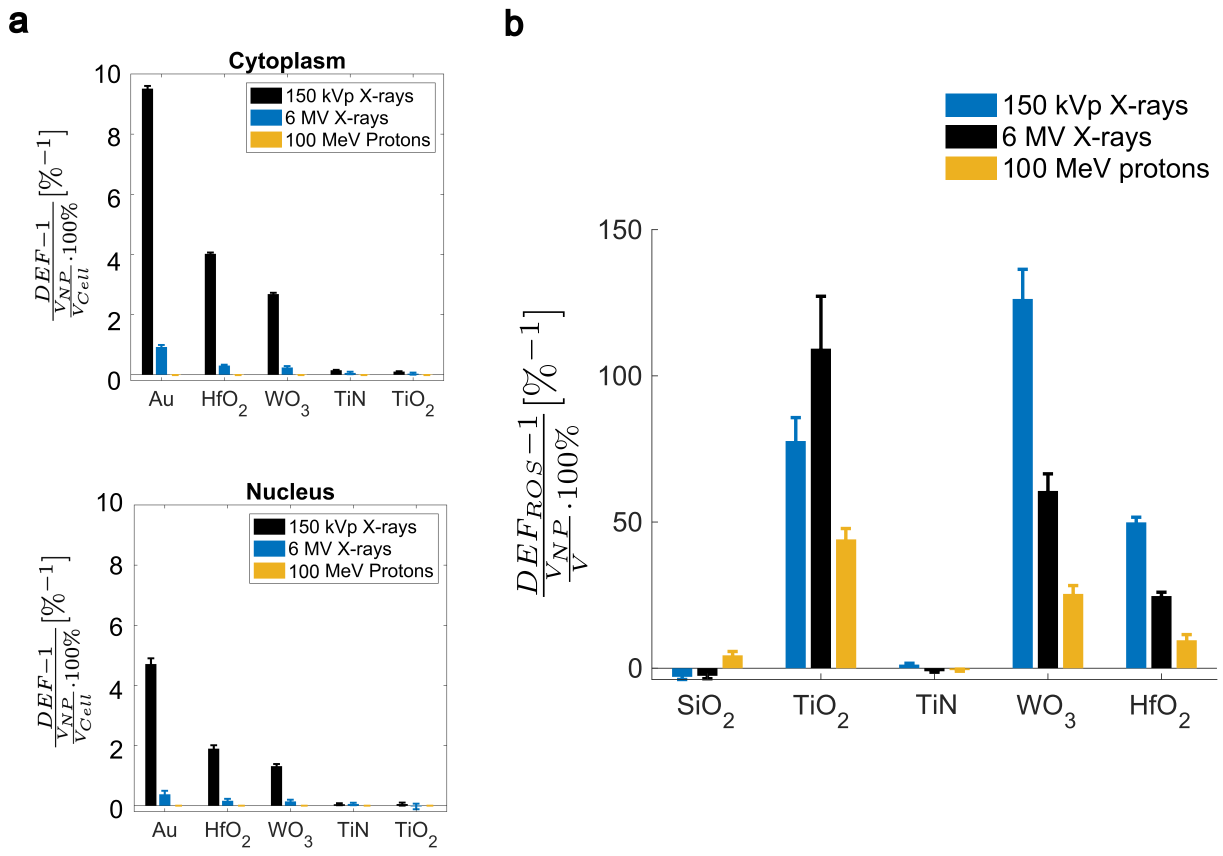

Supplementary Figure 5: Physical and chemical dose enhancement per nanoparticle volume fraction. Comparison of the linear regression coefficients of the physical dose enhancement factor (DEF) (a) and the chemical ROS dose enhancement factor (DEF_{ROS}) (b) per nanoparticle volume fraction in the cellular (cytoplasm and nucleus) and acellular system, respectively. Data expressed as linear regression coefficient ± 95%CI (in a: data from 3 simulation experiments with n = 12 sample points per regression; in b: data from 2 experiments with n = 18 sample points per regression).

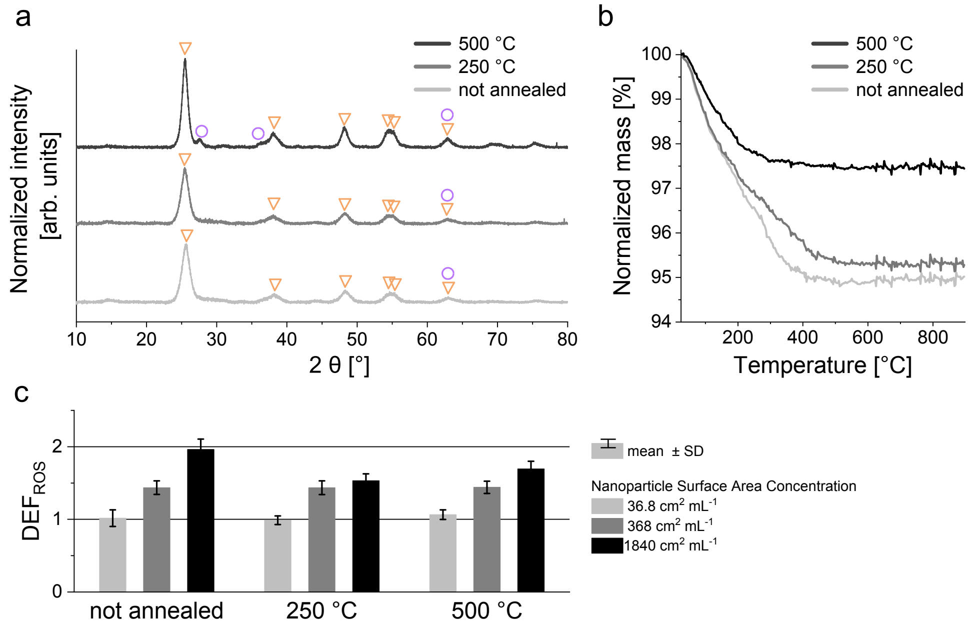

Supplementary Figure 6: Effect of annealing temperature on crystallinity, organic surface residues and radical generation under 150 kVp photon irradiation. XRD patterns (a), TGA (b) and chemical dose enhancement factor (DEF_{ROS}) (c) of as synthesized (not annealed), 250°C and 500°C annealed FSP made TiO_2 nanoparticles. In a: Orange open triangles denote anatase, while purple open circles denote rutile crystal phase. Data in c given as mean ± SD from n = 3 replicates.

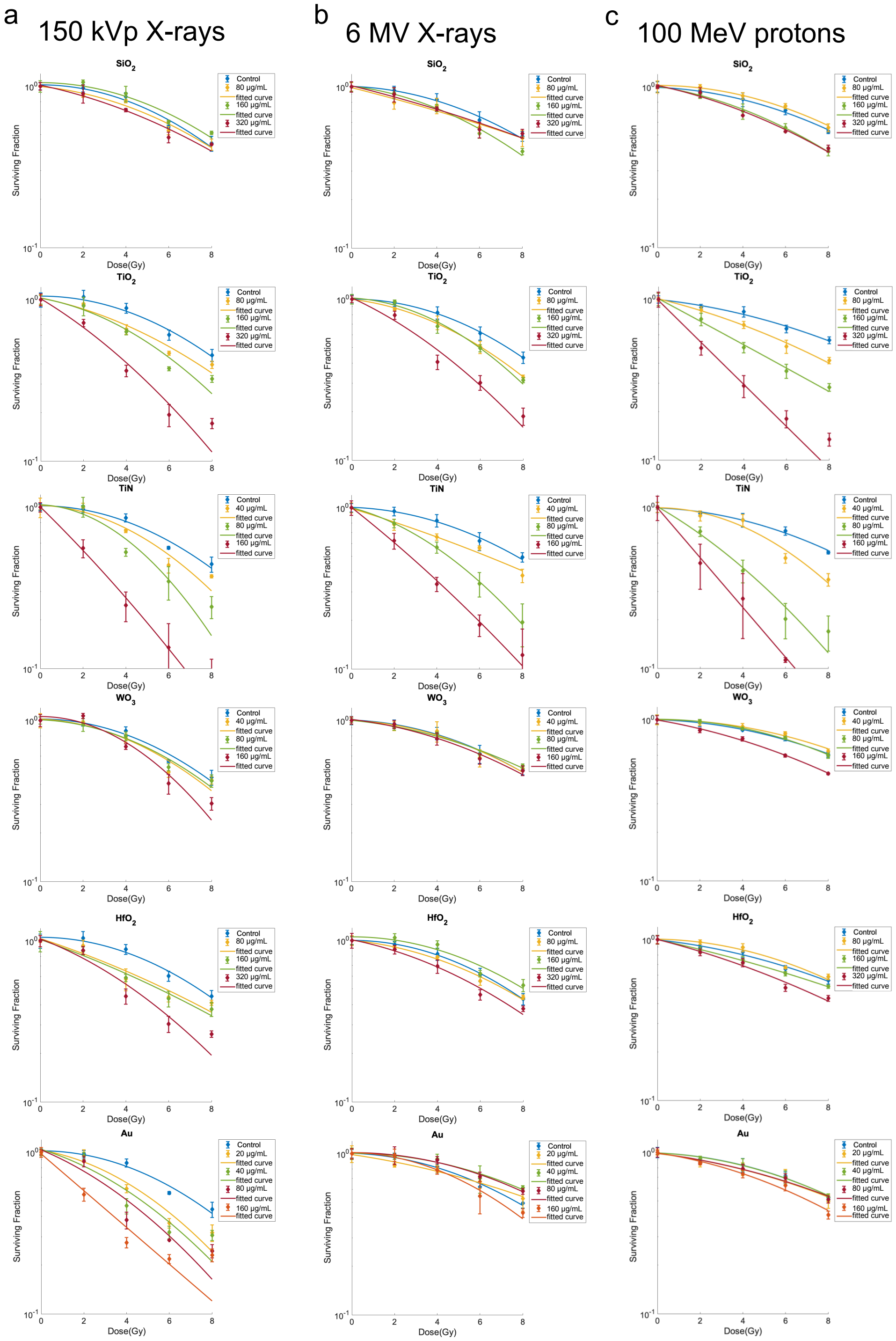

Supplementary Figure 7: In-vitro nanoparticle dose enhancement. Surviving curves and linear quadratic survival fits of HT1080 cells incubated with different concentrations of nanoparticles (yellow, green, red and orange data points and curves) or without nanoparticles (control, blue data points and curve) after irradiation with different doses of 150 kVp (a), 6 MV (b) or 100 MeV (c) X-rays or protons. Data expressed as mean ± SD and linear quadratic survival curve fit (straight line) exemplary for one experiment with n = 3 replicates per X-ray dose.

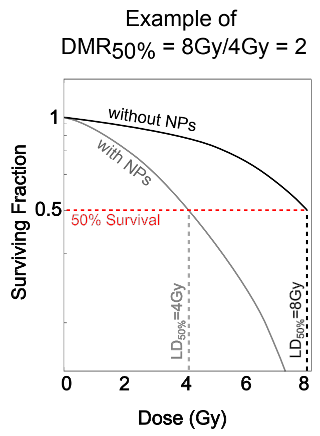

Supplementary Figure 8: Schematic representation of the extraction of the Dose Modifying Ratio ($DMR_{50\%}$) from cell survival curves. The $DMR_{50\%}$ is calculated by dividing the radiation doses (in Gy) without versus with nanoparticles which led to the same effect of 50% cell survival.

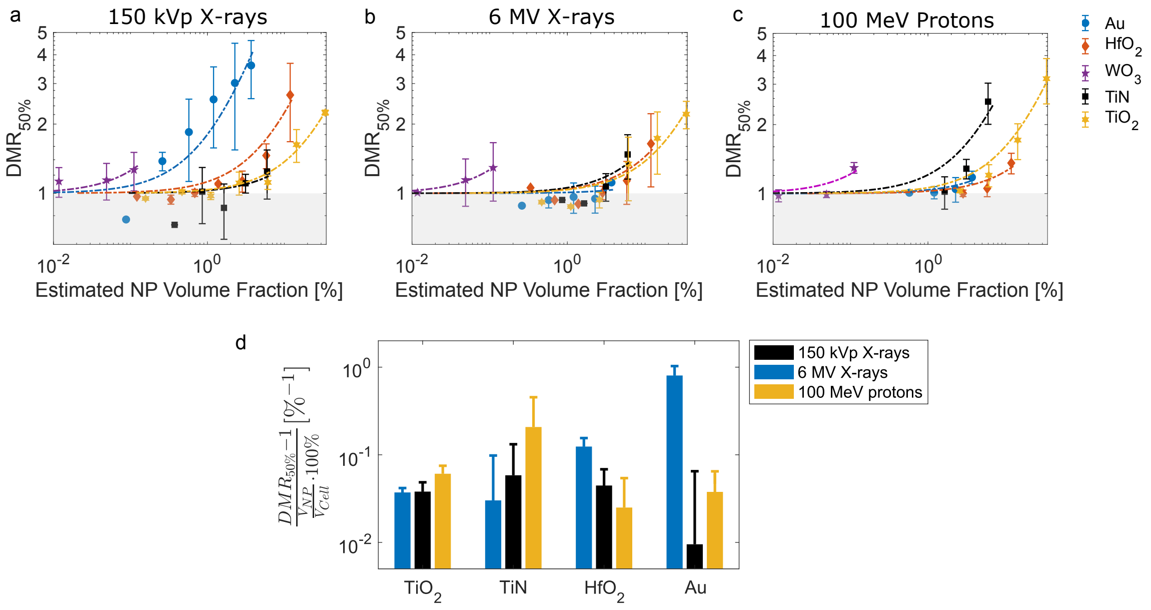

Supplementary Figure 9: Radioenhancement effects in HT1080 cells in relation to nanoparticle (NP) volume fraction per cell. Dose Modifying Ratio at 50% cell survival ($DMR_{50\%}$) (mean ± SD) and linear fit (dashed line) versus Au, HfO_2, WO_3, TiN and TiO_2 nanoparticle uptake under 150 kVp (a) and 6 MV X-rays (b) as well as 100 MeV proton (c) irradiation. Linear regression coefficients of the $DMR_{50\%}$ versus the nanoparticle volume fraction with 95% confidence interval (coefficient ± 95%CI) (d). Data in a-c derived from survival curves with n = 15 independent biological samples (3 samples per x-ray dose) and expressed as mean ± SD from at least 2 independent cell batches. The sample size n for the regression in d was 14, 14 and 10 for TiO_2, 9, 7 and 10 for TiN, 14, 11 and 8 for HfO_2 and 18, 10 and 14 for Au nanoparticles and kVp X-rays, MV X-rays and MeV protons, respectively.

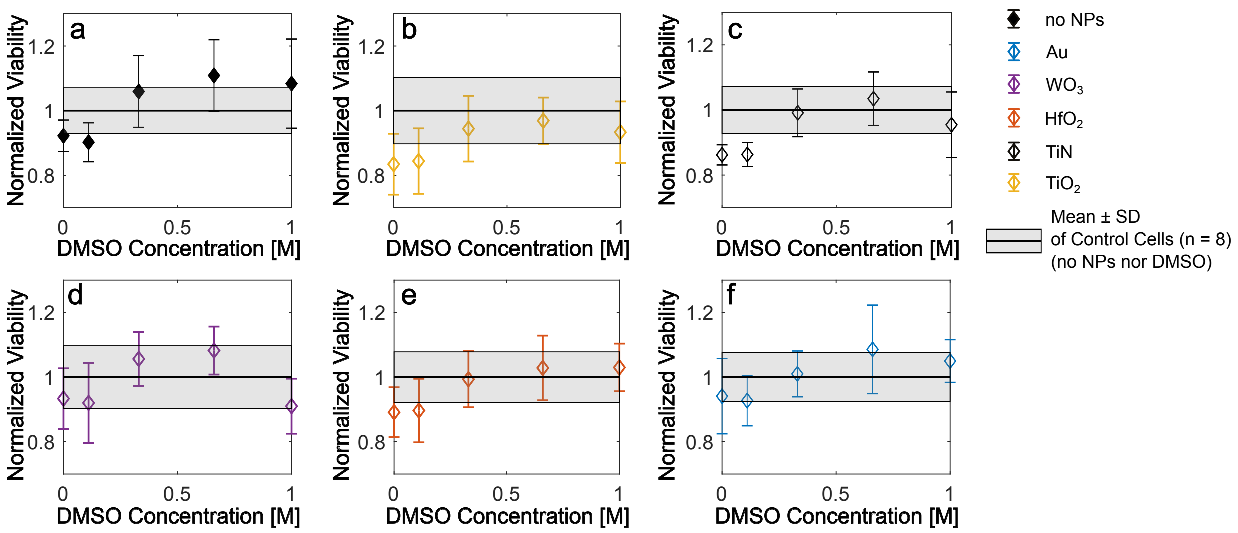

Supplementary Figure 10: Effect of short DMSO treatment on long term cell viability. Sham irradiation (0 Gy) viability of HT1080 cells for all nanoparticles (NPs) with and without DMSO treatment normalized to control cells (no DMSO, no nanoparticles). Cells have been pre-incubated with nanoparticle vehicle (10% MilliQ water) (a), 160 µg/mL TiO_2 (b), 80 µg/mL TiN (c), 160 µg/mL WO_3 (d), 320 µg/mL HfO_2 (e) and 40 µg/mL Au nanoparticles (f) for 24 h before DMSO and X-ray treatment; data analyzed as mean ± SD from n = 8 biological replicates examined over 2 independent experiments.

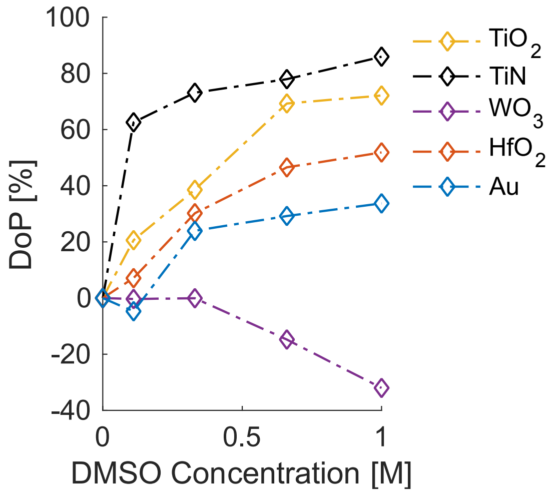

Supplementary Figure 11: Average degree of protection (DoP) of the nanoparticle dose enhancement by DMSO in dependence on DMSO concentration for all nanoparticles used. Data presented as mean values (n = 8). DMSO protects cell damage by quenching •OH radicals produced during irradiation.

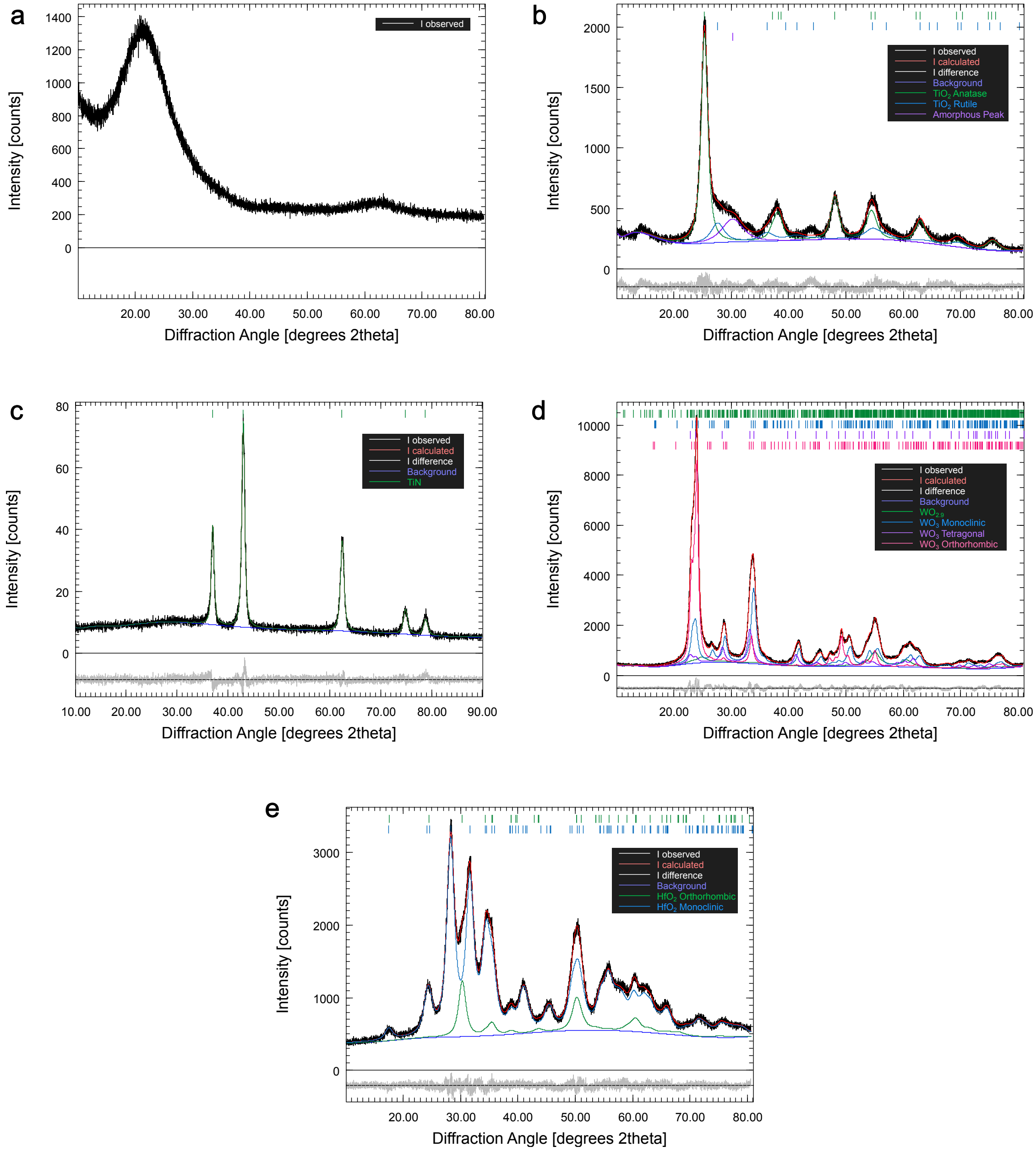

Supplementary Figure 12: Nanoparticle crystallinity. XRD patterns and Rietveld refinement for SiO_2 (a), TiO_2 (b), TiN (c), WO_3 (d) and HfO_2 (e) nanoparticles. For all crystalline samples (not SiO_2) resulting χ^2 and goodness of fit (GOF) were χ^2=1.67 and GOF=1.29 for TiO_2, χ^2=0.06 and GOF=0.25 for TiN, χ^2=3.0 and GOF=1.73 for WO_3 and χ^2=1.11 and GOF=1.05 for HfO_2.

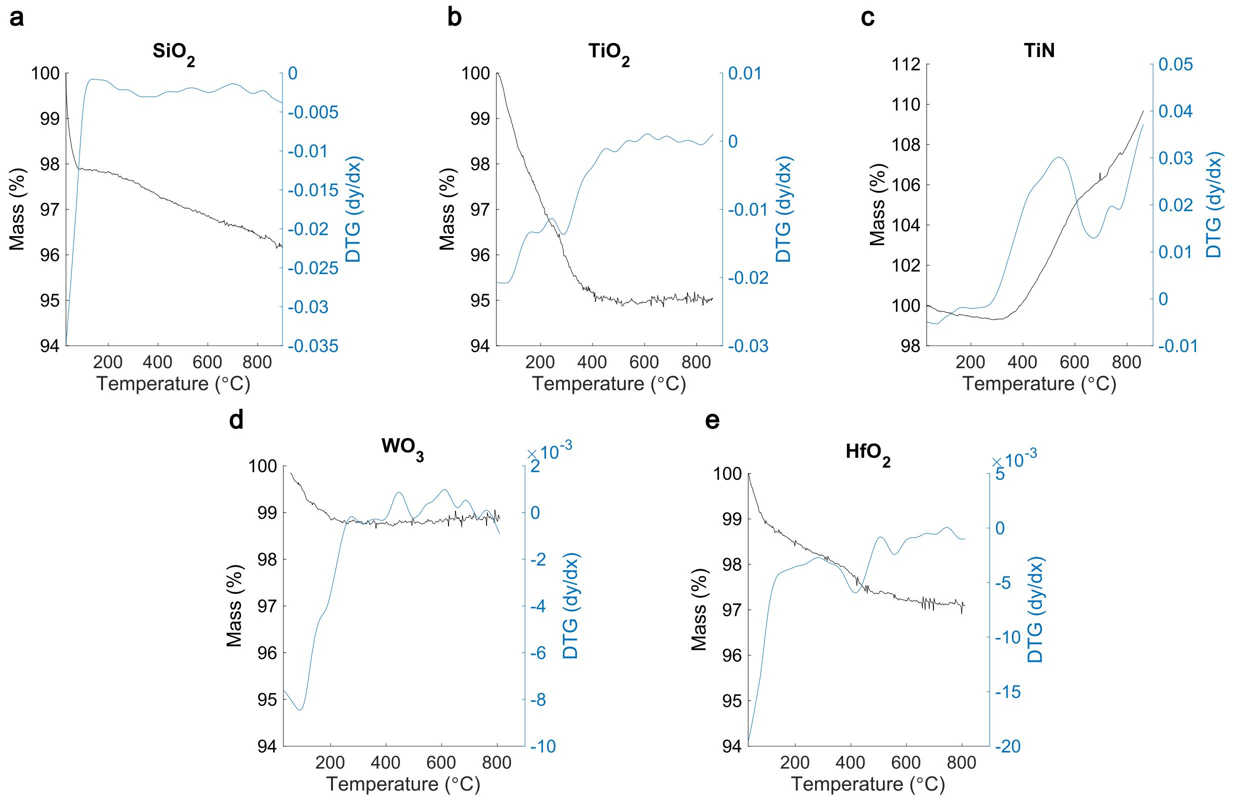

Supplementary Figure 13: Nanoparticle surface residue quantification. Thermogravimetric analysis (TGA, left y-axis, black) and its derivative (DTG, right y-axis, blue) for SiO_2 (a), TiO_2 (b), TiN (c), WO_3 (d) and HfO_2 (e) nanoparticles. The first mass loss (up to around 115°C) corresponds to loss of water. At higher temperatures organic residues leave. In c, weight loss up to ca. 350°C, thereafter weight gain due to nitrogen incorporation.

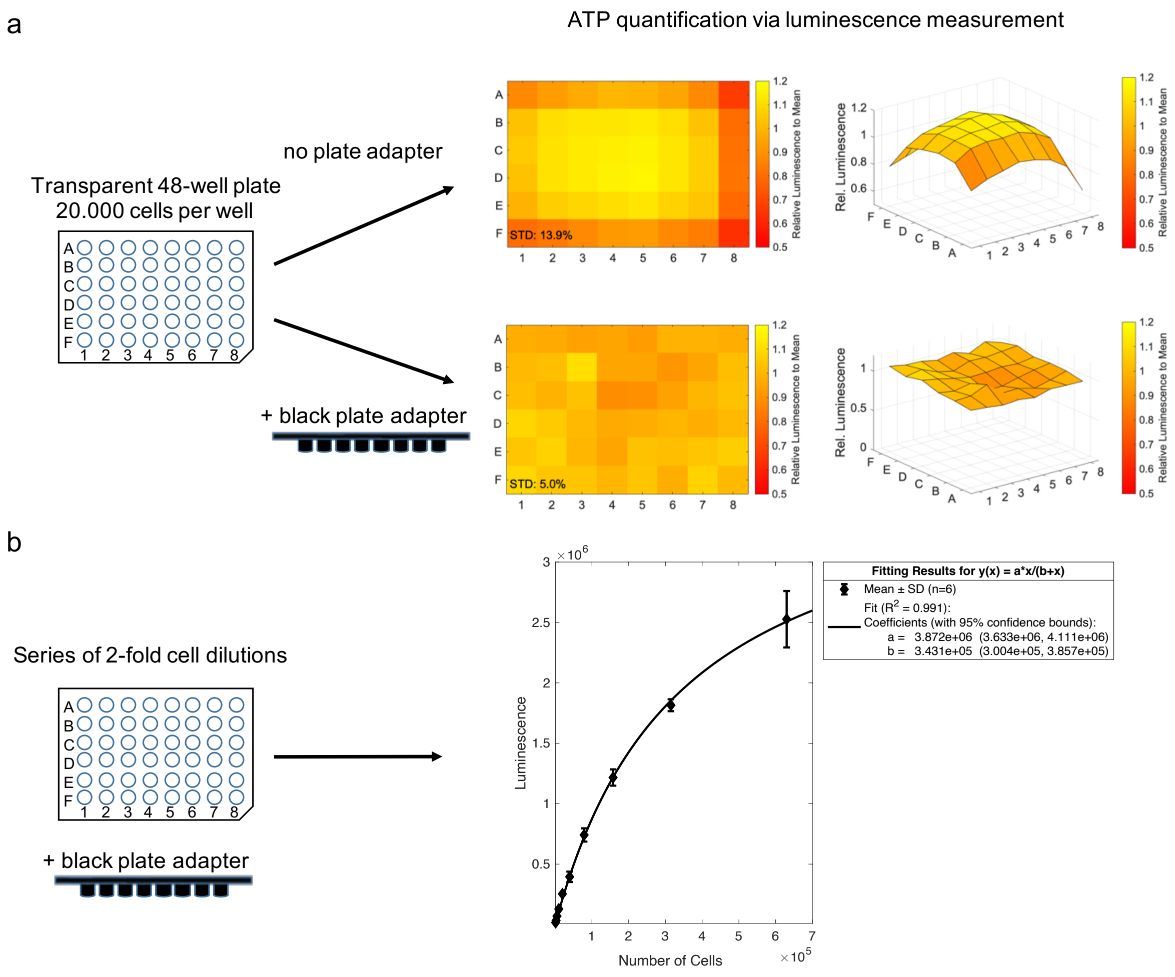

Supplementary Figure 14: Evaluation of black adapter plates to assess CellTiterGlo® luminescence from ATP conversion and cell number quantification. Without the adapter plates luminescence readings in transparent 48-well plates suffered from cross-talk, while the addition of adapter plates led to a homogenous luminescence reading across wells (a); STD: standard deviation of luminescence readings of all wells per plate. Cell standard curve showing the CellTiterGlo® luminescence per cell; data are presented as mean values ± SD from n = 6 biological replicates examined over 3 independent experiments (b).

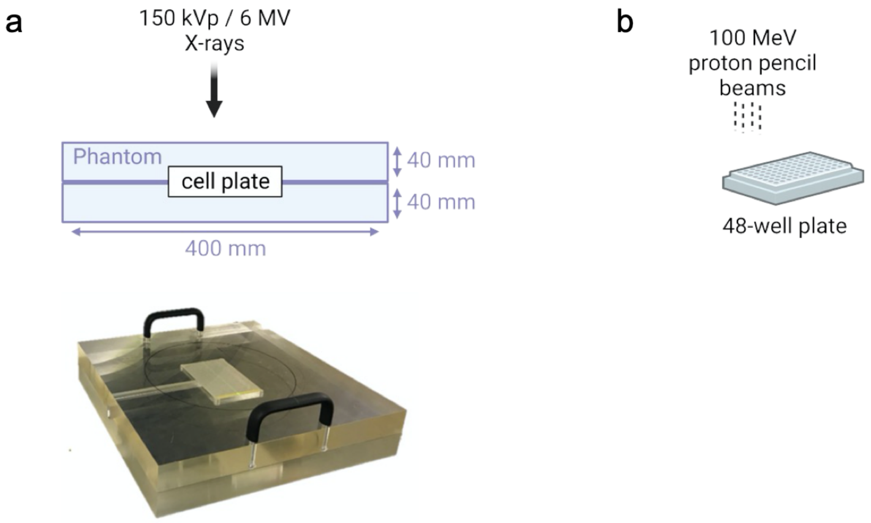

Supplementary Figure 15: Irradiation setup. Simple schematics of the irradiation setup geometry and picture of the PMMA phantom holding the cell plate for 150 kVp and 6 MV photon irradiation (a). Schematics of the irradiation set up for proton treatment (b). Partly created with BioRender.com.
